# Embedding implementation research to cross the quality of care chasm during the covid-19 pandemic and beyond

**DOI:** 10.1136/bmj-2023-076331

**Published:** 2023-12-11

**Authors:** Michael A Peters, Keith Cloete, George Odwe, Gizachew Tadele, Lisa R Hirschhorn, Hema Magge, Sanam Roder-DeWan

**Affiliations:** 1 World Bank Group, Washington DC, USA; 2 Western Cape Department of Health and Wellness, Cape Town, South Africa; 3 Population Council-Kenya, Nairobi, Kenya; 4 JSI Research and Training Institute, Addis Ababa, Ethiopia; 5 Northwestern University, Evanston, USA; 6 Bill and Melinda Gates Foundation, Seattle, USA; 7 Harvard University, Brigham and Women’s Hospital, Division of Global Equity, Boston, USA; 8 Dartmouth University, Hanover, USA

## Abstract

**Michael Peters and colleagues** argue that concerted efforts to embed implementation research can improve health services, even in the most challenging operating environments

As countries emerge from the acute phase of the SARS-CoV-2 (covid-19) pandemic, there is a need to reflect on global systems of knowledge production and uptake. The rapidly evolving context of the covid-19 pandemic response demanded timely interpretation and translation of evidence into policy and action to reduce morbidity and mortality, yet existing systems were largely unable to keep up with demand.^1^ Decision making informed by health services research was essential during this time of flux but is also important for continuous improvement of quality healthcare delivery. Before 2020, poor quality healthcare contributed to more lives lost in low and middle income countries (LMICs) than lack of access to health services.^2^ The size of the gap between the care patients should receive and the care patients actually receive was characterised in the American health system as a “quality chasm” as early as 2001.^3^ This chasm also exists globally: the Lancet global health commission on high quality health systems found that poor quality care is common across countries and is preventing people from realising optimal health.^4^ ^5^ The covid-19 pandemic further widened the quality chasm, emphasising the need for strategies to improve delivery of health services. 2 6 7

In a research environment that historically prioritises the development of new technologies or interventions over improving the delivery of existing interventions, emphasis should be placed on strategies that enable health research to improve implementation.^8^ Delivering evidence based health interventions with a minimum acceptable level of quality to improve outcomes is particularly challenging, making quality improvement a prime target for implementation research.^9^ ^10^ Bridging this gap is achieved by understanding what works in “real world” settings and paying specific attention to the end users of research.^9^ ^10^ Despite these principles, implementation research studies are often not done under real life management conditions, decision makers are not effectively engaged in the research process, and communication of research activities and results are not straightforward.^11^ ^12^ Embedding implementation research enables researchers and decision makers (policy makers and implementers) to operate in a connected system where research is an integrated and systematic part of operations, with continuous roles for policy makers, implementers, and communities to collaborate.^13^ ^14^ Embedding researchers tackles traditional implementation research shortcomings by placing decision makers at the centre of the research process and building the research and learning capacity of organisations and health systems.

Models of embeddedness of implementation research depend on the relationships between researchers, policy makers, and implementers during the study process.^15^ Embeddedness can occur at various levels of the health system: at the individual level through researchers integrated into service delivery systems; at the programmatic level, where research and learning collaborations are tied to specific health project cycles; and at the organisational level through integration into formal planning and budgeting processes (fig 1).^16^ ^17^ ^18^ The effectiveness of embedding implementation research at any level may be limited by stakeholder priorities and capacity to absorb information or by researcher ability to provide actionable findings.^19^

**Fig 1:**
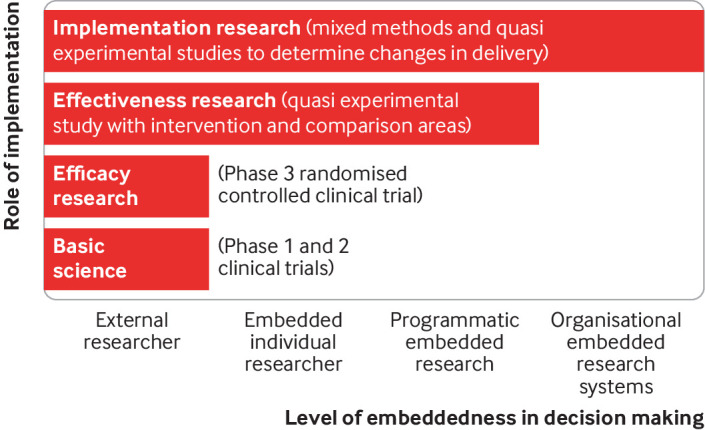
Levels of embeddedness in research

Embedding implementation research can strengthen the communication between researchers and decision makers, and they can move towards consensus on learning agendas and co-production of knowledge. However, barriers for embedding implementation research reported in studies in LMICs include the low baseline appreciation of research from stakeholders, lack of timeliness, and difficulties with political processes and organisational cultures.^20^ ^21^

In this analysis, we argue that concerted efforts to embed implementation research through organisational or pragmatic approaches can improve the impact of research on health services in LMICs, even in the most challenging operating environments, using the covid-19 response as a case study.

## Embedding implementation research in organisations to increase responsiveness

During the early stages of the covid-19 pandemic, governments that had teams of trained and trusted researchers were better equipped to implement evidence based policy to maintain high quality care during the response.^22^ One such example, the Western Cape Province in South Africa, has a history of partnerships with academic institutions, including several staff who hold joint positions with the provincial department of health and a local university, and routine processes for research priority setting, review, and analysis.^23^ Public health was strengthened in this partnership with the establishment of a dedicated directorate in 2012. This system of learning, supported through existing systems of embedded implementation research, better positioned the health system to adapt to evolving evidence and ensure high quality care during the response to the covid-19 pandemic.^24^ Health systems can become learning organisations by crystallising lessons and ensuring that learning is integrated within decision making processes.^25^ Experiences from South Africa demonstrate how embedding implementation research can support cycles of preparedness, response, and recovery to improve health system responsiveness to health shocks.

In South Africa’s Western Cape Province, pre-existing systems for embedding implementation research supported the development of clinical guidelines, interventions, and evidence based public health measures that improved health systems performance. In addition to the organisational culture of the health department, access to and use of a real time, trusted, patient level information database was instrumental in creating an effective system-wide response. Frequent “huddles,” or action orientated engagements with key evidence providers (academic partners) and stakeholders (Department of Health planners), were held throughout the early acute stages of the pandemic. Real time information on patient risk factors, covid-19 diagnosis, and outcomes provided analysts with data to respond to decision makers’ needs, which in turn enabled them to enact appropriate policy and strategies. More importantly, the same systems of data collection and review enabled decision makers to quickly identify when outcomes were worsening or becoming less equitable over time, prompting a quick response. After showing that outcomes in patients with covid-19 were worse during periods when health services were under the greatest pressure from covid-19 admissions, Western Cape Department of Health and Wellness implemented public health and social measures to reduce hospital service pressure and measured their effects. These strategies took the form of firstly, reducing covid-19 transmission through lockdowns, and secondly, reducing trauma admissions (non- covid-19 health service pressure) through an alcohol ban to free up capacity to care for covid-19 patients.^26^ Importantly, multisectoral partnerships and data collection and use capacities were in place before the pandemic, giving the province flexibility to respond to a wide range of emerging problems.^27^ A flexible warning system was established, matching levels of disease transmission with public health and social measures to ensure that care of covid-19 patients was not compromised by overcrowded hospitals.^28^ The evidence based and transparent warning system helped to set expectations with health system users and adapted service delivery systems.^29^

## Programmatic approaches for embedding implementation research to improve service quality

Programmatic approaches to embedding implementation research entail local researchers working closely with decision makers and implementers to embed research, evidence, and evaluation into routine implementation cycles of programmes or projects, rather than changing organisational cultures.^17^ Such initiatives have been supported by a range of donors and global health actors, but few have described their impact during the covid-19 pandemic.^12^ ^21^ ^30^ ^31^ In several countries, programmatic implementation research was embedded into existing newborn health programmes to improve the diagnosis and treatment of possible serious bacterial infection (PSBI) in children during the covid-19 pandemic.

PSBI is a major cause of infant mortality in sub-Saharan Africa, responsible for 37% of infant deaths each year.^29^ Despite the demonstrated efficacy of simplified treatment regimens, diagnosis and effective management of PSBI continues to be low owing to poor implementation of guidelines, resulting in unnecessary deaths.^5^ ^32^ ^33^ Using different embedded implementation research models, teams in Ethiopia and Kenya strengthened the implementation of PSBI protocols during the covid-19 pandemic despite facing increased challenges due to the pandemic.

In Ethiopia, the Addis Ababa based research team embedded with both the federal Ministry of Health and woreda (district) level health officials to develop and then contextualise PSBI implementation strategies for two woredas. Decision makers prioritised the implementation challenges for further research, identified potential solutions and strategies, and facilitated the implementation, review, and adaptation of strategies.^34^ During the implementation phase, data were collected through routine supportive supervision visits, shared with the research team, and then synthesised along common implementation research frameworks and shared with decision makers to inform needed adaptations. Local communities of practice were established to support across site learning, which were then relayed to higher level ministry officials. This co-creation of knowledge resulted in early buy-in from ministry officials to carry out several procedural changes to tackle specific implementation problems. In one district, new data collection and decision support processes were integrated into existing routine data systems; and in another district, training, supportive supervision, and supply chain management were strengthened to tackle management gaps identified during implementation. At the federal ministry level, results and learning from the PSBI programme fed into larger discussions about how to improve integrated community case management nationwide. Embedded research helped to inform real time implementation strategies at the local government level, which ultimately influenced integration of PSBI treatment into federal child health programmes.

Researchers from Population Council-Kenya provided embedded implementation research support to government partners and implementers in two counties (Busia and Migori) to improve PSBI detection and management. As in Ethiopia, policymakers and implementers provided context specific guidance on the research questions of interest, beginning with a baseline formative assessment. Based on analysis of referral pathways collected during routine monitoring and supervision visits, the research team identified weak links between communities and health facilities as a major barrier to effective treatment of PSBI. The monthly assessment cycles brought together embedded researchers, implementers, and ministry officials and provided opportunities for real time learning and adaptation of interventions. This manifested in revising PSBI management protocols to account for covid-19 related restrictions, introducing digital support tools, and training community health workers to strengthen continuity of care. 

Despite initial concerns about the project’s longevity, the PSBI package was integrated into routine essential newborn health services in both counties. While both Ethiopia and Kenya had similar roles for researchers, policy makers, and implementers, the stakeholders concerned varied based on context (for example, both public and private sector involvement in Kenya and federal ministry officials in Ethiopia), and existing coordination mechanisms were repurposed and strengthened to improve timely uptake of research findings.

## Call to action

It is important to note that embeddedness can occur in varying degrees, and demand for such arrangements should ultimately come from decision makers themselves.^35^ ^36^ The upfront time cost to find the right partnership, decide on a model of embeddedness, and build trust may be worth the long term benefits of improved health system responsiveness and data driven decision making demonstrated in the Western Cape Province.^25^ Another consideration relates to ethical and empirical concerns stemming from the embedded researcher’s ability to maintain objectivity.^20^ Competencies for embedded implementation researchers have been previously described, and these arrangements can be more productive if clear guidelines about the role of the researcher is agreed on across all parties from the beginning of the engagement.^17^ ^19^ ^37^ There are hurdles to overcome, including additional development of ethical frameworks for conducting implementation research in LMICs, but embedding is a promising strategy for increasing the uptake and policy relevance of global health research.^38^ ^39^

Future health systems need to tackle today’s issues while also considering how to sustain these gains in the event of health emergencies like the covid-19 pandemic. Embedding implementation research is effective in improving delivery of life saving interventions while also building resilience to health threats. All parties in global health have roles to play: donors can include embeddedness as a criterion for funding, academics can advocate for and incentivise partnerships, and decision makers can integrate embedding into their organisations and health systems. Appropriate application of these approaches can support the incremental gains in intervention effectiveness required to continue improving population health and strengthening health systems.

**Key messages**

Embedding implementation research can improve health outcomes by ensuring research is an integrated and systematic part of health systems functions
Embedded implementation research can occur across multiple levels, including the organisational level through integration into formal planning and budgeting processes, and the programmatic level, where research and learning collaborations are tied to specific health project cycles
Embeddedness can prepare health systems to respond to emerging health shocks like the covid-19 pandemic and improve the quality of service delivery, even in the most challenging operating environments
Donors, policymakers, implementers, and researchers all have critical part to play in prioritising embeddedness and strengthening the research and learning capacity of health systems

## Notes

Contributors and sources: MAP is a health systems and policy researcher and a consultant at the World Bank. KC is the head of the Western Cape Department of Health and Wellness and led covid-19 response. GO is a research associate at Population Council-Kenya and manages projects on embedded implementation research. GT is the director of Monitoring, Evaluation, and Research and leads implementation research activities at JSI Research and Training Institute, Inc. LRH is the senior director of implementation and improvement science at Last Mile Health and a professor of medical social sciences at North western University. HM is a senior programme officer at the Bill and Melinda Gates Foundation. SRD is a family physician and associate professor of community and family medicine. MAP wrote the first draft. KC, GO, GT, LRH, HM, and SRD reviewed and revised subsequent drafts. Peer reviewed literature, grey literature, and author experience were used to write this manuscript. SRD is the guarantor.

Provenance and peer review: Commissioned; externally peer reviewed.

This article is part of a collection proposed by the World Health Organization and the World Bank and commissioned by *The BMJ*. *The BMJ* peer reviewed, edited, and made the decision to publish these articles. Article handling fees are funded by the Bill and Melinda Gates Foundation. Jennifer Rasanathan, Juan Franco, and Emma Veitch edited this collection for *The* *BMJ*. Regina Kamoga was the patient editor.
